# Stress hyperglycaemia in critically ill patients and the subsequent risk of diabetes: a systematic review and meta-analysis

**DOI:** 10.1186/s13054-016-1471-6

**Published:** 2016-09-27

**Authors:** Yasmine Ali Abdelhamid, Palash Kar, Mark E. Finnis, Liza K. Phillips, Mark P. Plummer, Jonathan E. Shaw, Michael Horowitz, Adam M. Deane

**Affiliations:** 1 Intensive Care Unit, Royal Adelaide Hospital, North Terrace, Adelaide, SA 5000 Australia; 2 Discipline of Acute Care Medicine, The University of Adelaide, Adelaide, SA 5005 Australia; 3 Discipline of Medicine, The University of Adelaide, Adelaide, SA 5005 Australia; 4 Endocrine and Metabolic Unit, Royal Adelaide Hospital, North Terrace, Adelaide, SA 5000 Australia; 5 Intensive Care Unit, Addenbrooke’s Hospital, Hills Road, Cambridge, CB2 0QQ UK; 6 Clinical Diabetes Laboratory, Baker IDI, 75 Commercial Road, Melbourne, VIC 3004 Australia

**Keywords:** Critical care, Blood glucose, Hyperglycaemia, Type 2 diabetes mellitus, Prediabetes, Meta-analysis

## Abstract

**Background:**

Hyperglycaemia occurs frequently in critically ill patients without diabetes. We conducted a systematic review and meta-analysis to evaluate whether this ‘stress hyperglycaemia’ identifies survivors of critical illness at increased risk of subsequently developing diabetes.

**Methods:**

We searched the MEDLINE and Embase databases from their inception to February 2016. We included observational studies evaluating adults admitted to the intensive care unit (ICU) who developed stress hyperglycaemia if the researchers reported incident diabetes or prediabetes diagnosed ≥3 months after hospital discharge. Two reviewers independently screened the titles and abstracts of identified studies and evaluated the full text of relevant studies. Data were extracted using pre-defined data fields, and risk of bias was assessed using the Newcastle-Ottawa Scale. Pooled ORs with 95 % CIs for the occurrence of diabetes were calculated using a random-effects model.

**Results:**

Four cohort studies provided 2923 participants, including 698 with stress hyperglycaemia and 131 cases of newly diagnosed diabetes. Stress hyperglycaemia was associated with increased risk of incident diabetes (OR 3.48; 95 % CI 2.02–5.98; *I*^*2*^ = 36.5 %). Studies differed with regard to definitions of stress hyperglycaemia, follow-up and cohorts studied.

**Conclusions:**

Stress hyperglycaemia during ICU admission is associated with increased risk of incident diabetes. The strength of this association remains uncertain because of statistical and clinical heterogeneity among the included studies.

**Electronic supplementary material:**

The online version of this article (doi:10.1186/s13054-016-1471-6) contains supplementary material, which is available to authorized users.

## Background

‘Stress hyperglycaemia’ is defined as a blood glucose concentration that, in health, would lead to a diagnosis of diabetes [1–3] and represents a state of temporary insulin resistance and concomitant relative insulin deficiency [4, 5]. While stress hyperglycaemia is associated with greater illness severity and short-term mortality [2, 6, 7], it typically resolves, at least acutely, following recovery [8]. For this reason, stress hyperglycaemia has traditionally not been considered to have an adverse impact on long-term health. It is plausible, however, that critical illness uncovers latent insulin resistance and/or impaired pancreatic β-cell function, such that stress hyperglycaemia identifies patients at risk of subsequently developing diabetes [9].

Transient hyperglycaemia occurring in other contexts of physiological ‘stress’, such as pregnancy, is known to predict the development of diabetes [10–12]. Post-partum screening programmes for women with gestational diabetes allow early identification of type 2 diabetes to delay or reduce the associated complications [13–15].

The impact of stress hyperglycaemia on the risk of incident diabetes for survivors of critical illness remains unclear. We therefore performed a systematic review and meta-analysis of observational studies to evaluate the longitudinal risk of developing diabetes in critically ill patients with stress hyperglycaemia. Our secondary objective was to evaluate the impact of stress hyperglycaemia on the risk of prediabetes (impaired fasting glucose and/or impaired glucose tolerance).

## Methods

We performed this meta-analysis in accordance with the Meta-analysis of Observational Studies in Epidemiology (MOOSE) statement [16]. Methods and inclusion criteria were specified and documented in advance (Additional file 1).

### Eligibility criteria

Eligible studies met the following criteria: (a) retrospective or prospective controlled study design (case-control or controlled cohort), (b) study population of adult patients (aged ≥18 years) admitted to an intensive care unit (ICU), (c) exposure to stress hyperglycaemia with normoglycaemia during ICU admission as the reference exposure and (d) outcomes of development of diabetes or prediabetes diagnosed ≥3 months after ICU discharge. Studies that reported a diagnosis of diabetes only at ICU admission or shortly after ICU discharge (within 3 months) were excluded, as they were deemed to be reporting rates of established but previously undiagnosed diabetes [2]. Studies that reported outcomes for acutely ill patients not admitted to an ICU were excluded. In studies with overlapping samples, we included only the largest study to avoid duplication of data. We considered only studies reported in English. No date or publication status restrictions were imposed.

### Data sources and searches

A librarian and two reviewers (YA and PK) searched the MEDLINE and Embase databases (from their inception to February 2016). Searches included synonyms and combinations of the following terms: ‘critical illness’, ‘intensive care’, ‘hyperglycaemia’, ‘glucose’, ‘insulin’, ‘type 2 diabetes’ and ‘prediabetes’. Terms were truncated in order to capture variable terminology. The full search strategies are provided in Additional file 2. We applied no language restrictions during the searches. We also reviewed reference lists of retrieved papers to identify other potentially eligible studies not captured in the primary search.

### Study selection

Two reviewers independently screened titles and abstracts of all identified studies. Relevant studies were independently evaluated in full text for eligibility. Disagreements were resolved by consensus or by consultation with a third reviewer. In order to avoid duplications from several reports of the same study, a comparison was conducted across studies when needed, checking for authors, study locations, sample sizes and outcomes.

### Quality assessment

Two reviewers independently assessed methodological quality using the 8-item Newcastle-Ottawa Scale (NOS) [17]. Risk of bias was assigned on the basis of the number of NOS items deemed inadequate for each study: low risk of bias (0 or 1 item), medium risk of bias (two or three items), high risk of bias (more than three items) or very high risk of bias (no description of methods). Studies judged to be at high or very high risk of bias were excluded from the meta-analysis.

### Data extraction

Two reviewers independently extracted data from included studies using a standardized data collection form. Extracted information included study characteristics (author, publication year, country, design, sample size), participant characteristics (age, sex, diagnosis, illness severity, mortality, body mass index [BMI], family history of diabetes, steroid use, nutrition delivery), definition of stress hyperglycaemia and method of detection, methods to exclude pre-existing undiagnosed diabetes, definitions of diabetes and prediabetes, methods to diagnose diabetes or prediabetes, duration of follow-up, ORs for the development of diabetes and/or prediabetes with corresponding 95 % CIs, and any statistical adjustment performed for the competing risk of death.

The supplementary files of all included studies were also examined for the purposes of data extraction. When necessary, we contacted the authors of the included studies for additional information.

### Data synthesis and statistical analysis

The OR (95 % CI) was used as the measure of association between stress hyperglycaemia and the development of diabetes or prediabetes across the studies. We used the Cochran Q statistic (*p* < 0.1) and the *I*^2^ statistic to investigate the possibility of statistical heterogeneity [18]. Meta-analysis was performed using a random-effects model, and a pooled OR with 95 % CI was calculated. We elected a priori to perform an additional subgroup analysis of studies that excluded patients with pre-existing unrecognised diabetes on the basis of glycated haemoglobin (HbA1c) level on ICU admission [19]. As there were only a small number of studies, graphic representation of publication bias was not performed [20]. Analyses were performed using STATA version 14.1 software (StataCorp, College Station, TX, USA).

## Results

### Study selection

**Fig. 1:**
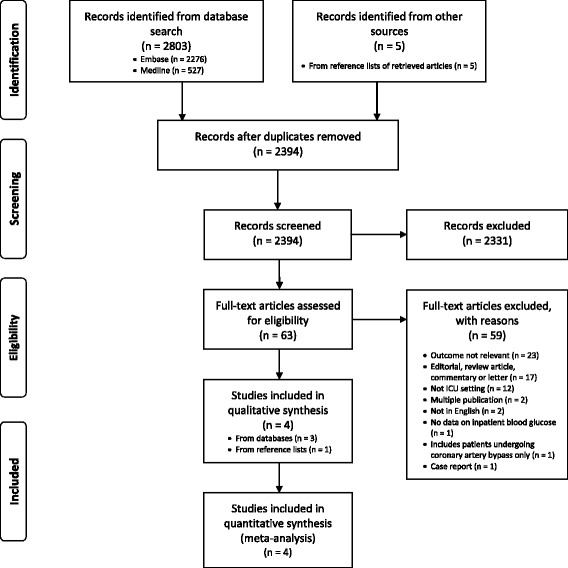
Flow diagram for selection of studies, *ICU* Intensive care unit

Our search yielded 2389 non-duplicate citations. We discarded 2331 (on the basis of title and abstract) because they did not meet the inclusion criteria. Five additional records were identified from reference lists of relevant retrieved articles, with 63 articles evaluated in full text. Of these, 18 were not controlled studies, 23 did not assess a relevant outcome, 12 were not conducted in an ICU setting, two were duplicate reports, two were not in English and one did not include data on inpatient blood glucose levels. One conference abstract was excluded because it reported solely patients after coronary artery bypass graft surgery, and it was not deemed representative of the majority of patients admitted to the ICU due to the elective nature of the surgery and its association with a short ICU stay. After these exclusions, four cohort studies remained and were included in the analysis (Fig. 1). Because of the overlapping duration of recruitment periods for two studies at one centre [21, 22], the primary author was contacted and confirmed that each cohort contained different study participants.

### Study characteristics and risk of bias within studies

The characteristics of the included studies [21–24] are summarised in Table 1. In three single-centre studies, researchers recalled patients after ICU discharge to test for diabetes or prediabetes with an oral glucose tolerance test (OGTT) [21, 22, 24]. Additionally, in one study, researchers performed HbA1c testing at ICU admission and 8 months after discharge, but this was not performed for all enrolled patients [24]. One study was a multi-centre database record linkage study evaluating the risk of diabetes in patients with stress hyperglycaemia who had emergency admissions to hospital [23]. Only the subgroup of patients admitted to the ICU in this study was included.

**Table 1:** Summary of included studies evaluating subsequent risk of diabetes in critically ill patients with stress hyperglycaemia

First author, year [reference]	Study design, location and recruitment period	Follow-up duration	Participants	Recruitment: total number (normal/SH); males %; age in years, median (IQR)	Follow-up: number completing; normal (%), SH (%)	SH definition	Nutrition	Number of new cases of diabetes; normal (%), SH (%)	Methods used to: (1) diagnose incident diabetes and (2) exclude baseline diabetes
Gornik, 2010 [21]	Single-centre, PC, Croatia, July 1998–June 2004	5 years	Medical patients with no history of steroid use, pancreatitis, disturbed glucose metabolism or other endocrine disorder who were admitted to ICU	1029 (669/360); 55 % males; age, normal 58 years (19–86), SH 59 years (22–87)	591; normal 398 (67 %), SH 193 (33 %)	Venous BG in ICU >7.7 mmol/L, measured twice per day with point-of-care blood gas analyser	EN and PN	47; normal 14 (4 %), SH 33 (17 %)	(1) Annual OGTT for 5 years^a^ (2) History; OGTT 4–6 weeks after discharge
Gornik, 2010 [22]	Single-centre, PC, Croatia, January 2000–December 2002	5 years	Patients admitted to ICU with sepsis, acute coronary syndrome and acute heart failure with no history of disturbed glucose metabolism or steroid use	258 (168/90); 54 % males; age, normal 57 years (48–65), SH 60 years (49–65)	166; normal 115 (69 %), SH 51 (31 %)	Random venous BG in ICU >7.7 mmol/L on at least two occasions	Not stated	12; normal 4 (3 %), SH 8 (16 %)	(1) OGTT: follow-up of at least 5 years but frequency not specified^a^ (2) History; absence of hyperglycaemia before discharge
McAllister, 2014 [23]	Multi-centre, RC, Scotland, December 2004–November 2008	3 years	Patients aged ≥30 years with an emergency admission to hospital between 2004 and 2008^b^	1828^b^; sex and age not specified for ICU subgroup	1828; normal 1620 (89 %), SH 208 (11 %)^b^	Admission BG (first BG taken within 2 days of admission) ≥11.1 mmol/L	Not stated	48; normal 37 (2 %), SH 11 (5 %)^*b*^	(1) Record of new diagnosis in national register^c^ between 31 days and 3 years after discharge (2) Record in national register^c^ prior to admission or within 30 days of discharge; admission BG >20 mmol/L
Van Ackerbroeck, 2015 [24]	Single-centre, PC, Belgium, September 2011–March 2013	8 months	Patients aged 18–85 years admitted to a medical-surgical ICU for ≥48 h; patients with pancreatitis, known disturbed glucose metabolism and those using glucose-lowering drugs excluded	385^d^; 66 % males; age, normal 56 years (18–82), SH 62 years (20–88)	338; normal 92 (27 %), SH 246 (73 %)	Arterial BG >140 mg/dl (>7.8 mmol/L) measured using on-site blood gas analyser	EN and PN	24; normal 4 (4 %), SH 20 (8 %)	(1) OGTT with or without HbA1c 8 months after ICU admission^a^ (2) History; medication review; with or without HbA1c^e^

*Abbreviations: PC* Prospective cohort, *RC* Retrospective cohort, *ICU* Intensive care unit, *SH* Stress hyperglycaemia, *BG* Blood glucose, *EN* Enteral nutrition, *PN* Parenteral nutrition, *OGTT* Oral glucose tolerance test, *HbA1c* Glycated haemoglobin, *IQR* interquartile range

^a^Diabetes defined according to American Diabetes Association criteria: fasting plasma glucose ≥7.0 mmol/L or 2-h plasma glucose ≥11.1 mmol/L during a 75-g OGTT performed as described by the World Health Organisation or HbA1c ≥6.5 % (48 mmol/mol) [19]

^b^Only the subgroup of 1828 patients admitted to ICU is included in the analysis. The total number of patients included in the study is 86,634

^c^Scottish Care Information-Diabetes Collaboration is a national register including >99 % of people with diabetes in Scotland

^d^Number of patients with normoglycaemia and stress hyperglycaemia in original cohort not stated

^e^Admission HbA1c measured in only 45 % of study population. HbA1c ≥6.5 % (48 mmol/mol) considered diagnostic of diabetes

In total, 2923 ICU survivors from four studies were included. Illness severity was inconsistently reported. Only one study reported ventilation rates and provided illness severity scores [24]. Three studies defined stress hyperglycaemia as ≥7.8 mmol/L. The database linkage study used a higher threshold (≥11.1 mmol/L) [23]. The relationship between the timing of blood glucose measurement and the delivery of nutrition was not reported in any study. Three studies [21, 22, 24] defined diabetes and prediabetes according to published consensus criteria for plasma glucose and HbA1c [19]. The database linkage study [23] determined incident diabetes following registration with the national register.

The risk of bias within included studies is presented (Table 2). Three studies [21, 22, 24] were deemed to be at risk of incomplete outcome data due to the number and limited description of patients lost to follow-up. One study provided no description of whether missing outcome data were equal across the stress hyperglycaemia and normoglycaemia cohorts [24]. In general, stress hyperglycaemia and normoglycaemia cohorts were comparable in terms of age, sex and, when reported, nutrient delivery. However, when reported, the stress hyperglycaemia cohorts had a higher BMI, more frequent family history of diabetes and greater illness severity. No data on the specific characteristics of the subgroup of patients admitted to ICU in the database linkage study [23] were provided. Finally, each study employed different methods to identify patients with pre-existing undiagnosed diabetes (Table 1). No study was deemed at overall high or very high risk of bias, and therefore all four studies were included in the meta-analysis.

**Table 2:** Risk of bias within included studies assessed using the Newcastle-Ottawa Scale

First author, year [reference]	Selection (maximum score 4⋆)				Comparability of cohorts (maximum score 2⋆)	Outcome (maximum score 3⋆)			Total score, risk of bias
	Representativeness of the exposed cohort	Selection of non-exposed cohort	Ascertainment of exposure	Demonstration that outcome of interest was not present at start of study		Assessment of outcome	Duration of follow-up	Adequacy of follow-up	
Gornik, 2010 [21]	⋆	⋆	⋆	⋆	⋆	⋆	⋆	–	7, medium risk of bias
Gornik, 2010 [22]	⋆	⋆	⋆	–	⋆	⋆	⋆	–	6, medium risk of bias
McAllister, 2014 [23]	–	⋆	⋆	⋆	–	⋆	⋆	⋆	6, medium risk of bias
Van Ackerbroeck, 2015 [24]	⋆	⋆	⋆	⋆	⋆	⋆	–	–	6, medium risk of bias

### Stress hyperglycaemia and the risk of diabetes

**Fig. 2:**
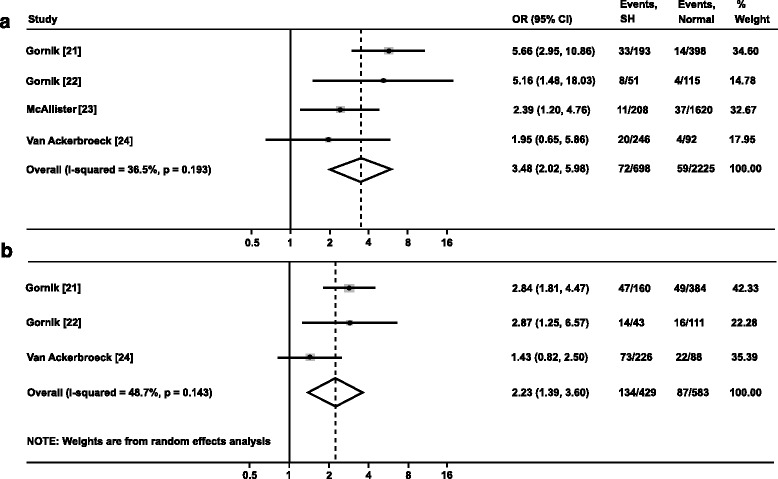
**a** Forest plot showing the risk of diabetes in critically ill adult patients with stress hyperglycaemia. **b** Forest plot showing the risk of prediabetes in critically ill adult patients with stress hyperglycaemia. *SH* stress hyperglycaemia. Prediabetes was defined according to American Diabetes Association criteria: fasting plasma glucose 5.6–6.9 mmol/L (impaired fasting glucose), or 2-h plasma glucose during 75-g oral glucose tolerance test 7.8–11.0 mmol/L (impaired glucose tolerance), or glycated haemoglobin 5.7–6.4 % (39–46 mmol/mol) [19]

Among the 2923 participants, 698 (23.9 %) experienced stress hyperglycaemia and 131 (4.5 %) cases of incident diabetes were detected during follow-up. Stress hyperglycaemia was associated with an increased risk of developing diabetes in survivors of critical illness, with low to moderate degrees of heterogeneity between studies (Fig. 2a).

No studies measured HbA1c levels on ICU admission for the majority of patients, so we were unable to perform our pre-specified subgroup analysis. We were unable to undertake further subgroup analyses to examine the effects of age, sex and diagnosis because of the small number of events and inconsistent reporting of this information.

### Stress hyperglycaemia and the risk of prediabetes

Three studies [21, 22, 24] reported risk of developing prediabetes, defined according to the same criteria [19]. Among the 2923 participants, 221 (7.6 %) cases of prediabetes were detected during follow-up. Stress hyperglycaemia was associated with increased risk of developing prediabetes in survivors of critical illness, with a moderate degree of heterogeneity between studies (Fig. 2b).

## Discussion

### Main findings

We undertook the first meta-analysis to examine the impact of stress hyperglycaemia in survivors of critical illness. Our findings suggest that stress hyperglycaemia identifies patients at increased risk of incident diabetes. In addition, stress hyperglycaemia also identified patients at increased risk of developing prediabetes, a well-accepted risk factor for type 2 diabetes with an annual conversion rate in ambulatory subjects of 5–10 % [25]. Our observations are consistent with outcomes of other studies performed in non-ICU settings including patients following stroke [26], myocardial infarction [27, 28] and pneumonia [29] where comparable rates of incident diabetes following stress hyperglycaemia were observed.

### Clinical implications

Our findings have substantial clinical significance. There usually exists a protracted period of time between the development of diabetes and its diagnosis, with microvascular complications often established at the time of diagnosis [30]. If stress hyperglycaemia during critical illness identifies a population at risk of diabetes, an opportunity exists for early diagnosis and intervention to prevent long-term complications of diabetes. Readily available and cost-effective strategies, such as the use of metformin and lifestyle interventions including weight loss and exercise, exist to reduce progression to diabetes in at-risk populations. These strategies have been demonstrated to be effective in patients with prediabetes and in women with prior gestational diabetes [15, 31–33].

While general population screening programmes for type 2 diabetes are not always cost-effective [34], targeted screening of high-risk groups, as is the case in gestational diabetes, improves health outcomes [35]. Our meta-analysis suggests that the risk of diabetes in ICU survivors with stress hyperglycaemia is similar to the risk in women with gestational diabetes over comparable observation periods [10, 12]. Furthermore, survivors of critical illness often experience long-term physical problems [36–38] and therefore may have a unique capacity to benefit from screening programmes to identify prediabetes or diabetes.

### Potential mechanisms

Failure of pancreatic β-cells to meet insulin secretory demand in the face of diminished insulin sensitivity is fundamental to the pathogenesis of type 2 diabetes [39]. Several mechanisms appear to underlie stress hyperglycaemia during critical illness, including increased release of counter-regulatory hormones, altered insulin receptor signalling due to inflammation, pancreatic β-cell inhibition and interventions such as administration of glucocorticoids or parenteral nutrition [1, 8, 40]. However, the studies included in our meta-analysis also reported that, in patients with stress hyperglycaemia, there was more often a family history of diabetes and higher BMI, suggesting that well-accepted risk factors for diabetes also contribute to the development of stress hyperglycaemia. Mechanistically, it is highly plausible that one or more pre-existing disorders of insulin sensitivity and/or production result in predisposition to stress hyperglycaemia during critical illness and may lead to subsequent development of diabetes. We also speculate that additional mechanisms may be implicated in the progression to diabetes in survivors of critical illness. These include the reduction in physical activity post-ICU [37] and autonomic dysfunction, which affects more than half of ICU patients [41].

### Strengths and limitations

Strengths of our meta-analysis include the structured search, complete retrieval of the identified research and validated methods in accordance with the MOOSE statement. Included cohort studies were of reasonable methodological quality, particularly given the logistical challenges involved in studying these cohorts, and almost 3000 patients were included.

However, our study has limitations. We included only studies in English. We were also unable to exclude publication bias, and negative studies may be missing, potentially resulting in overestimation of the effect size. Our meta-analysis reflects data derived from only four studies, which limits our certainty in the results [42]. In addition, along with moderate statistical heterogeneity, we observed considerable clinical heterogeneity between the studies; for example, definitions of stress hyperglycaemia, methods of outcome assessment and duration of follow-up differed.

Conceptually, stress hyperglycaemia is defined by a glucose concentration normally indicative of diabetes (i.e., random blood glucose ≥11.1 mmol/L). However, a strict definition has not been consistently applied, and whether a single elevated reading is sufficient or documentation of more than one episode of hyperglycaemia is required has yet to be established. Given that there were no corresponding data identifying that blood glucose concentrations were fasting or post-prandial, three studies [21, 22, 24] used a relatively low threshold for stress hyperglycaemia (≥7.8 mmol/L), which could underestimate the risk of diabetes. Conversely, the study which utilised a threshold of ≥11.1 mmol/L [23] required only a single elevated reading, which may not be sufficiently specific to identify risk, because transient disturbances in blood glucose can occur during critical illness following administration of catecholamines or corticosteroids. Furthermore, only two studies specifically excluded patients who received corticosteroids [21, 22].

Overall, the small number of incident events (diabetes) in our meta-analysis means that our point estimates have greater uncertainty [43] and that our ability to assess the effects of age, sex and diagnosis on risk of diabetes is limited. In addition, some patients with undiagnosed diabetes may not have been recognised at baseline and could have been misclassified as incident diabetes cases. These patients would have been more likely categorised in the stress hyperglycaemia group, and this differential misclassification could bias toward inflating the estimates of risk for incident diabetes. Only one study formally tested all patients with an OGTT to exclude pre-existing diabetes [21]. However, gastric emptying is delayed during critical illness [44], and gastric emptying is a major determinant of oral glucose tolerance in health and diabetes [44, 45]. This has implications for the interpretation of the OGTT, such that identification of unrecognised diabetes using the OGTT in critically ill patients is uncertain. None of the studies measured HbA1c on admission for the majority of patients. HbA1c is a validated tool for the diagnosis of previously unrecognised diabetes in hospitalised and critically ill patients [46–48], and consensus guidelines now recommend the measurement of HbA1c in all hospitalised patients with hyperglycaemia [49].

Individual study results were also likely influenced by management of missing data. Most studies had high rates of withdrawal, and limited descriptions were provided of patients lost to follow-up. It is plausible that patients lost to follow-up were those who experienced greater illness severity and subsequent impaired mobility. These patients may have a higher risk of disturbed glucose metabolism, and the true incidence of diabetes may have been underestimated. It is also possible that patients who develop hyperglycaemia during ICU admission are likely to receive more intense screening for diabetes after hospital discharge than those who remained normoglycaemic throughout their ICU admission [49]. Furthermore, in one study, the duration of follow-up was short (8 months), and the risk of incident diabetes may increase with period of observation [24]. Across the four studies included in our meta-analysis, the OR for incident diabetes was observed to increase with increasing duration of follow-up. Only one study performed statistical adjustment for the competing risk of death [23].

There are also limitations on the generalisability of individual study results, for the following reasons: information about illness severity is absent in most studies, only a small subset of patients was admitted to the ICU in the large multi-centre study [23], two single-centre studies [21, 22] included a high proportion of patients presenting with myocardial ischaemia, and one study reported high rates of parenteral nutrition administration [21]. We restricted our search to studies of patients admitted to the ICU, and our results may not reflect outcomes of acutely ill patients not admitted to the ICU. Furthermore, the two studies that demonstrated the strongest relationship between stress hyperglycaemia and subsequent incident diabetes [21, 22] were conducted in the same centre, and this is a limitation of our findings. However, it is important to note that these studies had the longest duration of follow-up and were the only studies to recall patients regularly after ICU discharge and formally test for diabetes.

### Implications for research

Our meta-analysis supports the concept that stress hyperglycaemia is a risk factor for incident diabetes in survivors of critical illness. A multi-centre, prospective cohort study with a follow-up period of several years would be required to precisely quantify this risk. Such a study should define stress hyperglycaemia on the basis of repeated blood glucose measurements and in relation to nutrient delivery, as well as utilise routine measurement of HbA1c to exclude undiagnosed diabetes at baseline. Furthermore, studies which evaluate mechanisms underlying progressive glucose intolerance are required because such understanding is critical to guiding intervention.

## Conclusions

Stress hyperglycaemia during ICU admission is associated with increased risk for incident diabetes. The strength of this relationship should be interpreted with caution because of statistical and clinical heterogeneity among the included studies.

## Additional files

Additional file 1: Review protocol. (DOCX 18 kb) Additional file 2: Search strategies. (DOCX 17 kb)

## Abbreviations

BG: Blood glucose
BMI: Body mass index
CI: Confidence intervals
EN: Enteral nutrition
HbA1c: Glycated haemoglobin
ICU: Intensive care unit
MOOSE: Meta-analysis of Observational Studies in Epidemiology
NOS: Newcastle-Ottawa Scale
OGTT: Oral glucose tolerance test
OR: Odds ratios
PC: Prospective cohort
PN: Parenteral nutrition
RC: Retrospective cohort
SH: Stress hyperglycaemia
