# Supplementary material for: Stress hyperglycaemia in critically ill patients and the subsequent risk of diabetes: a systematic review and meta-analysis
Source: Crit Care. 2016 Sep 27;20:301. doi: 10.1186/s13054-016-1471-6 (PMC5039881; doi:10.1186/s13054-016-1471-6)
Supplement: Additional file 1: — Review protocol. (DOCX 18 kb) [file 13054_2016_1471_MOESM1_ESM.docx]

Additional file 1 Review Protocol

Title

Stress Hyperglycaemia in Critically Ill Patients and the Subsequent Risk of Diabetes: A Systematic Review and Meta-Analysis

Hypothesis

Our primary hypothesis is that stress hyperglycaemia during admission to Intensive Care Unit (ICU) identifies those survivors of critical illness with substantially increased risk for the subsequent development of type 2 diabetes.

Our secondary hypotheses are that:

1. Stress hyperglycaemia during admission to ICU identifies those survivors of critical illness with substantially increased risk for the subsequent development of prediabetes
2. The risk of diabetes and prediabetes increases with duration of follow up
3. The risk of diabetes and prediabetes depends on admission diagnosis (cardiovascular > other)
4. The association between diabetes and prediabetes is independent of age
5. The association between diabetes and prediabetes is independent of sex

Methods of Review

The study will be conducted according to the PRISMA statement.

*Data Sources*

Two electronic databases (Medline, EMBASE) will be searched with the assistance of a Health Sciences librarian. Search strategy to be developed in conjunction with Health Sciences librarian to include terms relating to intensive care and critical illness, hyperglycaemia, prediabetes and diabetes.

Reference lists of retrieved articles will be reviewed to identify additional articles for possible inclusion.

Contact with authors if necessary.

*Inclusion Criteria*

Study design: controlled (case-control or controlled cohort), retrospective or prospective, no limits on year or publication status

Study population: critically ill adult patients (>18 years) admitted to an ICU

Exposure: stress hyperglycaemia in ICU; accept a range of definitions of stress hyperglycaemia

Comparison: critically ill adult patients with normoglycaemia in ICU

Outcome: diabetes or prediabetes reported at least 3 months after ICU discharge

Outcome measurement: all methods of diagnosis to be considered

*Exclusion Criteria*

Study in a language other than English.

Studies reporting on an acutely ill population not admitted to an intensive care unit (e.g. admitted to a general medical or surgical ward, cardiac ward).

Studies reporting on diabetic/prediabetic status only during ICU admission or shortly after discharge (within 3 months), using oral glucose tolerance test (OGTT) or glycated haemoglobin (HbA1c), with no other follow up of patients.

*Assessment for Eligibility and Data Extraction*

To be performed independently by two reviewers (YA and PK). Disagreements resolved by consensus or by consulting with a third reviewer (AMD).

Data extraction to be done independently by two reviewers (YA and PK) using a standardised data extraction sheet which will include the study characteristics and the results of interest listed below.

Authors to be contacted for clarification if needed.

Study Characteristics and Results of Interest for Data Extraction

*Population*

Screened for prediabetes or diabetes on admission to ICU (HbA1c)?

Excluded patients on steroids?

Severity of illness (APACHE II or other, how many ventilated, days in ICU, mortality in ICU or hospital?)

Demographics on admission (age, sex, body weight, family history of diabetes) recorded and presented?

Admission diagnoses

Threshold for ‘stress hyperglycaemia’ (single or repeated)

Threshold fasted or fed (assume fed if not identified)

If not screened on admission were patients screened for prediabetes or diabetes soon after discharge from ICU (e.g. 30 days or 3 months) and removed from analysis?

*Comparator*

Comparator group (ICU patients without stress hyperglycaemia)

Screened for prediabetes or diabetes on admission to ICU

Demographics on admission (age, sex, body weight, family history of diabetes) recorded and presented? (matched to stress hyperglycaemia group)

Demographics on admission (age, sex, body weight)

Admission categories

*Outcomes*

Primary outcome: Diabetes (Definition used, repeated or single test, included all of fasting blood glucose, OGTT and HbA1c?)

Secondary outcome: Prediabetes (Definition used, repeated or single test, included all of fasting blood glucose, OGTT and HbA1c?)

Duration of time between ICU admission and testing (maximum follow up, frequency of follow up)

Measured on site or used other marker

Effect size and 95% CI

*Setting*

Single or multicentre

Country of origin (background prevalence)

Type of ICU (tertiary/academic or other, Medical/Surgical/Mixed)]

*Description of study design*

Prospective or retrospective

Statistics – Do studies adjust for competing risk of death?

Numbers loss to follow up/reported loss to follow up

Risk of Bias

Two reviewers (YA and PK) to assess for risk of bias with Newcastle-Ottawa Quality Assessment Scale. Risk of bias to be judged based on consensus with arbitration by third reviewer (AMD) if necessary. Studies at high risk of bias may be excluded from meta-analysis.

Statistical Analysis

Aggregate data to be used.

Pooled odds ratio or relative risk if possible. Important heterogeneity expected among the studies. Analyses will be performed with a random effects model. Between-study heterogeneity will be assessed using the Cochran Q and I^2^ statistics. Analyses will be performed using STATA, version 14.1 (Stata Corp).

Cumulative incidence if possible.

*a priori* – include a separate meta-analysis of only studies that excluded unrecognised diabetes on admission by testing for HbA1c.
