# Supplementary material for: Stress hyperglycaemia in critically ill patients and the subsequent risk of diabetes: a systematic review and meta-analysis
Source: Crit Care. 2016 Sep 27;20:301. doi: 10.1186/s13054-016-1471-6 (PMC5039881; doi:10.1186/s13054-016-1471-6)
Supplement: Additional file 2: — Search strategies. (DOCX 17 kb) [file 13054_2016_1471_MOESM2_ESM.docx]

Additional file 2 Search Strategies

Database: MEDLINE 1946 to present Incl. In-Process & Other Non-Indexed Citations (Ovid)

Date: February 22, 2016

| 1 | (Hyperglyc$ or Glucose or Insulin).mp. |
| --- | --- |
| 2 | (Type 2 adj2 diabet$).mp. |
| 3 | (Type ii adj2 diabet$).mp. |
| 4 | Diabetes Mellitus, Type 2/ |
| 5 | 2 or 3 or 4 |
| 6 | (Prediabet$ or Disturbed glucose metabolism or Impaired fasting glucose or Glucose intolerance or Glucose tolerance).mp. |
| 7 | 5 or 6 |
| 8 | ((((Critical adj3 care) or Intensive) adj3 care) or Burn$ unit$ or Coronary care unit$ or Respiratory care unit$ or Critical$ ill$ or Multiple organ failure$ or ICU$).mp. |
| 9 | 1 and 7 and 8 |

[mp=title, abstract, original title, name of substance word, subject heading word, keyword heading word, protocol supplementary concept word, rare disease supplementary concept word, unique identifier]

Results: 527 references

Database: Embase 1974 to present (Ovid)

Date: February 22, 2016

| 1 | (Hyperglyc$ or Glucose or Insulin).mp. |
| --- | --- |
| 2 | exp Non insulin dependent diabetes mellitus/ |
| 3 | (Type 2 adj2 diabet$).mp. |
| 4 | (Type ii adj2 diabet$).mp. |
| 5 | Diabetes Mellitus, Type 2/ |
| 6 | Prediabet$.mp. |
| 7 | (Disturbed glucose metabolism or Impaired fasting glucose or Glucose intolerance or Glucose tolerance).mp. |
| 8 | 2 or 3 or 4 or 5 or 6 or 7 |
| 9 | (Critical adj3 care).mp. |
| 10 | (Intensive adj3 care).mp. |
| 11 | Burn$ unit$.mp. |
| 12 | Coronary care unit$.mp. |
| 13 | Respiratory care unit$.mp. |
| 14 | Critical$ ill$.mp. |
| 15 | Multiple organ failure$.mp. |
| 16 | ICU$.mp. |
| 17 | 9 or 10 or 11 or 12 or 13 or 14 or 15 or 16 |
| 18 | 1 and 8 and 7 |

[mp=title, abstract, original title, name of substance word, subject heading word, keyword heading word, protocol supplementary concept word, rare disease supplementary concept word, unique identifier]

Results: 2276 references
